# Ultrasound Irradiation Promoted Enzymatic Transesterification of (*R/S*)-1-Chloro-3-(1-naphthyloxy)-2-propanol

**DOI:** 10.3390/molecules170910864

**Published:** 2012-09-10

**Authors:** Feng Wang, Hong Zhang, Jiaxin Wang, Ge Chen, Xuedong Fang, Zhi Wang, Lei Wang

**Affiliations:** 1 Center of General Surgery, the Second Hospital of Jilin University, Changchun 130041, China; Email: wangfengdoctor@163.com (F.W.); fangxuedong@medmail.com.cn (X.F.); 2 Key Laboratory of Molecular Enzymology and Engineering of Ministry of Education, College of Life Sciences, Jilin University, Changchun 130023, China; Email: Zhanghong163163@163.com (H.Z.); jiaxin1012@163.com (J.W.); chengeilm4196@gmail.com (G.C.)

**Keywords:** propranolol, 1-chloro-3-(1-naphthyloxy)-2-propanol, lipase, transesterification, ultrasound

## Abstract

(*R*)-1-Chloro-3-(1-naphthyloxy)-2-propanol (**3**), which is the key intermediate of (*S*)-propranolol, was successfully prepared via enantioselective transesterification catalyzed by lipase under ultrasound irradiation. Compared with conventional shaking, the enzyme activity and enantioselectivity were dramatically increased under ultrasound exposure. Effects of various reaction conditions on the synthetic activity of enzyme as well as enantioselectivity, including the type of enzyme, ultrasound power, solvent, acyl donor, temperature and substrate molar ratio, were investigated. *Pseudomonas sp.* lipase (PSL) showed an excellent catalytic performance under optimum conditions (enzyme activity: 78.3 ± 3.2 μmol·g^−1^·min^−1^, *E* value: 98 ± 6).

## 1. Introduction

**Scheme 1:**
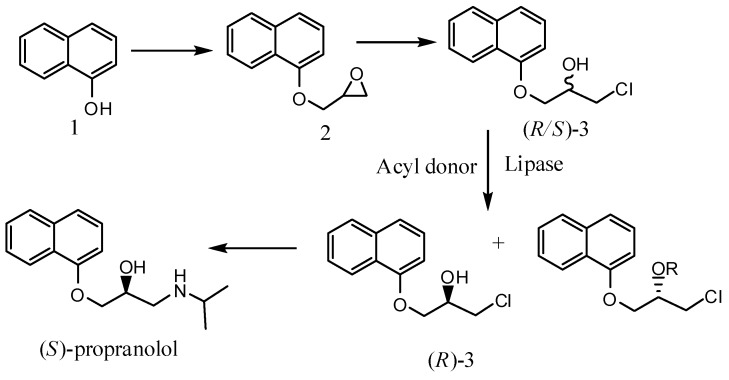
Chemo-enzymatic synthesis of (*S*)-propranolol.

Currently, the increasing understanding of the mechanisms of drug interactions on a molecular level has led to an increasing awareness of the importance of chirality as the key to the efficacy of many drug products [1]. It is now known that often only one enantiomer of a drug substance is required for efficacy, and the other enantiomer is either inactive or exhibits considerably reduced activity [2]. Propranolol is a β-blocker of the 3-(aryloxy)-l-(alkylamino)-2-propanol type, where the activity resides mainly in its *S* isomer. Moreover, (*R*)-propranolol could be used as a contraceptive [3]. Chiral drugs have been receiving more attention as a result of some studies relating those syntheses. One of the important ways is predicted to be through chemo-enzymatic synthesis. The widely use of lipase for production of enantiomeric products has been attempted as an efficient method, especially for producing high value-added chiral drug intermediates [4,5,6,7,8]. However, a major limitation in the enzymatic resolution of (*R*/*S*)-1-chloro-3-(1-naphthyloxy)-2-propanol (**3**), the key intermediate of propranolol, is the low reaction rate, which restricts the industrial production of enantiomeric propranolol (Scheme 1) [9].

It is well known that most lipases have a short α-helical “lid” covering the active center of the lipase [10]. Concerning the status of the lid and interfacial activation of lipases, the open-closed status of the conformation may be changed dramatically under various experimental conditions, which in turn strongly affect the activity and enantioselectivity of lipases [11]. Many methods (such as medium engineering, immobilization, chemical modification techniques, *etc*.) are often used to improve the performance of lipases [12,13,14]. Recently, ultrasound irradiation has been widely used to improve the reaction rates. Many interesting applications have been found in organic chemistry and biotechnology when ultrasound irradiation was used as an environmentally benign method [15,16,17]. The chemical and physical effects of ultrasound arise from the cavitational collapse that produces extreme local conditions and thus induces the formation of chemical species that are not easily achieved under conventional conditions, leading to a particular radical reactivity. The ultrasound irradiation can be satisfactorily applied in biocatalysis considering these cavitational collapse effects [18]. The enhanced effect of ultrasound is possibly due to acceleration of the collision probability of the enzyme and substrate. Meanwhile, cavitation also accelerates mass transport, so that product diffuses faster from the enzymatic site. Furthermore, smaller average size drops can be obtained and therefore, it increases the interfacial area and enzyme dispersion in this interface.

With all this in mind, the aim of this research was to investigate the effect of ultrasound irradiation on the lipase-catalyzed kinetic resolution of (*R*,*S*)-l-chloro-3-(l-napthyloxy)-2-propanol (**3**), and the optimization of the reaction conditions for this transformation.

## 2. Results and Discussion

### 2.1. Effect of Enzyme Type

It is well known that lipase catalytic transesterification depends mainly on the type and origin of the enzyme [19]. In this study, the effect of ultrasound on different enzyme-catalyzed reactions was investigated. Though conventional shaking may control the size of this aggregates and the dispersability of free lipase in organic solvent, ultrasound irradiation dramatically increased the reaction compared to the shaking method by reducing the mass transfer limitations. Furthermore, ultrasound irradiation could perturb weak protein interactions and induce its conformational change, which may improve some enzymatic reactions [20,21]. In view of the results shown in Table 1, CALB showed the highest enzyme activity, but PSL afforded the highest enantioselectivity and higher enzyme activity either under conventional shaking or ultrasound irradiation. *Pseudomonas* sp*.* lipase (PSL) displayed the characteristic of “pH memory” in organic media and was also thermostable in organic solvents with an optimum temperature range from 45 to 60 °C [22]. PSL is widely used in the resolution of racemic alcohols, thus PSL was chosen as the best enzyme source in the transesterification of (*R*,*S*)-l-chloro-3-(l-napthyloxy)-2-propanol (**3**).

**Table 1:** Effect of enzyme type on the enantioselective transesterification of alcohol **3**.

Enzyme	Conventional shaking		Ultrasound irradiation	
	Enzyme activity (μmol·g^−1^·min^−1^)	Enantioselectivity ( *E* value)	Enzyme activity (μmol·g^−1^·min^−1^)	Enantioselectivity ( *E* value)
*Pseudomonas sp.* Lipase (PSL)	43.3 ± 3.7	87 ± 7	78.3 ± 3.2	98 ± 6
*Candida antarctica* lipase B (CAL-B)	59.6 ± 2.3	70 ± 6	85.6 ± 3.1	72 ± 8
Porcine pancreas lipase (PPL)	23.7 ± 6.8	27 ± 4	36.7 ± 1.3	21 ± 2
*Candida sp. 415* lipase (CSL)	35.6 ± 4.4	34 ± 1	50.6 ± 2.5	36 ± 2
*Pseudomonas fluorescens* lipase (PFL)	29.8 ± 5.2	29 ± 2	41.8 ± 1.8	37 ± 3

The reactions were carried out in *n*-hexane (20 mL) with alcohol **3** (1 mmol), vinyl propionate (4 mmol) and enzyme (10 mg) at 45 °C. The ultrasound power was 175 W.

### 2.2. Effect of Ultrasound Power

**Figure 1:**
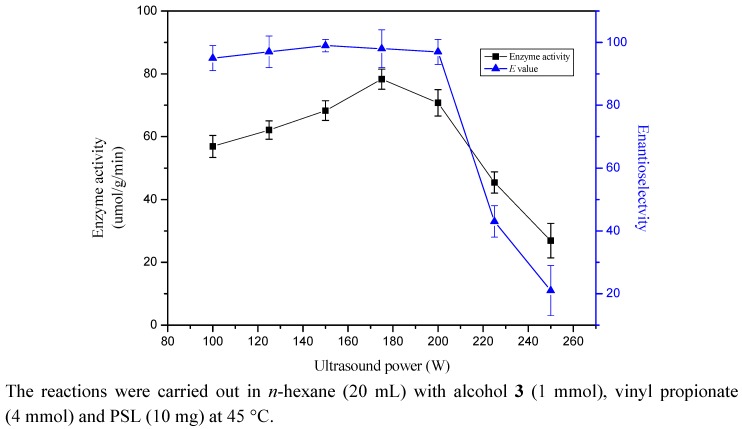
Effect of ultrasound power on the enantioselective transesterification of alcohol **3**.

Ultrasound power is an important influencing parameter for reactions under ultrasound irradiation. In this work, seven different 30 kHz ultrasound powers (100, 125, 150, 175, 200, 225, 250 W) were selected to examine the effect on the enantioselective transesterification of l-chloro-3-(l-napthyloxy)-2-propanol (**3**). As shown in Figure 1, the ultrasound power obviously affected the activity and enantioselectivity of the enzyme. The highest activity were obtained when the power = 175 W and a further increase in the ultrasound power resulted in an obvious decrease in enzyme activity (Figure 1). No significant change for the enantioselectivity of enzyme was observed when the power varied from 100-200 W, and was decreased marginally by further increasing the power. The enhancement of enzyme activity obtained at lower ultrasound power was probably attributed to a decrease in substrate inhibition and aggregation based on hydrogen bonding of molecules [23]. The enzyme conformation might be destroyed which occurred at higher ultrasonic power, then the enzymatic activity and enantioselectivity were decreased. This phenomenon was in accordance with the reports which state that the breakdown of the hydrogen bonding and van der Waals interactions in the protein could be due to ultrasound cavitation [24]. Furthermore, shear force which occurs in the reaction media during ultrasound irradiation may also play a significant role in enzyme inactivation [25]. Consequently we selected 175 W to study the characteristics of lipase-catalyzed transesterifications in the following experiments.

### 2.3. Effect of Solvent

Many researchers have reported that the organic medium influences not only the enzyme activity, but also the enantioselectivity [26]. In the present study, the effects of the solvents with different log *P* (logarithm of the partition coefficient of a given solvent between *n*-octanol and water; log *P* is widely used to denote the polarity or hydrophobicity of a solvent) were displayed in Table 2. The enzyme catalytic performance in organic solvents was significantly correlated with solvent hydrophobicity. The activity and enantioselectivity increased with the increasing of log *P* values. Solvents with low log *P* value, and thus more hydrophilic, tend to strip the essential bound water molecules present on the surface of the enzyme [27] causing a decrease in its catalytic activity. In addition, the strong interactions of hydrophilic solvent with enzyme resulted in the variation of the conformation of the enzyme active site and decreased the *E* values. From the solvents studied, *n*-hexane was selected as the appropriate solvent in the reaction.

**Table 2:** Effect of solvent on the enantioselective transesterification of alcohol **3**.

Solvent	Log *P*	Enzyme activity (μmol·g^−1^·min^−1^)	Enantioselectivity ( *E* value)
Acetonitrile	−0.33	10.9 ± 4.2	15 ± 2
Tetrahydrofuran	0.49	28.7 ± 2.4	27 ± 4
Cyclohexane	1.20	35.6 ± 3.2	53 ± 5
Toluene	2.50	61.4 ± 2.6	65 ± 3
Methyl *tert*-butyl ether	2.90	69.4 ± 1.3	90 ± 5
*n*-Hexane	3.50	78.3 ± 3.2	98 ± 6

The reactions were carried out in organic solvent (20 mL) with alcohol **3** (1 mmol), vinyl propionate (4 mmol) and enzyme (10 mg) at 45 °C. The ultrasound power was 175 W.

### 2.4. Effect of Acyl Donor

It is intriguing to note that the length of acyl chain strongly influences the resolution of a chiral alcohol. We selected five vinyl esters with different carbon chain lengths (C_2_-C_6_) as the acyl donors to carry out the transesterification. The results under ultrasound are presented in Table 3. The enzyme activity increased with decreasing chain length of the acyl donor, the activity being the highest for vinyl acetate. Acyl donors with short chain lengths might access the active pocket of the enzyme with greater ease. As for the enantioselectivity, the highest *E* value was found for vinyl propionate, as shorter or longer chains led to a decrease in the *E* value. It’s well known that PSL has a deep narrow active site [28]. The acyl and alcohol moieties are thereby brought close in space during catalysis, which could explain PSL’s sensitivity to the length of the acyl chain. Enantioselectivity mainly results from the disatereomeric interaction in the acyl-lipase-substrate complex. The acyl group of different vinyl esters transiently attached at the active center of the lipase participates in the alteration of the enantioselectivity as a stereochemical controller in the enantioselective transesterification [29]. Therefore, the weak interaction of acyl moiety of vinyl acetate with the active center of lipase may cause a lowest enantioselectivity in this transesterification. 

**Table 3:** Effect of acyl donor on the enantioselective transesterification of alcohol **3**.

Acyl donor	Vinyl acetate	Vinyl propionate	Vinyl butyrate	Vinyl valerate	Vinyl caproate
Enayzme activity (μmol·g^−1^·min^−1^)	89.6 ± 3.8	78.3 ± 3.2	66.1 ± 4.1	45.2 ± 3.0	20.4 ± 2.5
Enantioselectivity ( *E* value)	69 ± 2	98 ± 6	81 ± 3	73 ± 4	70 ± 5

The reactions were carried out in organic solvent (20 mL) with alcohol **3** (1 mmol), acyl donor (4 mmol) and enzyme (10 mg) at 45 °C. The ultrasound power was 175 W.

### 2.5. Effect of Temperature

**Figure 2:**
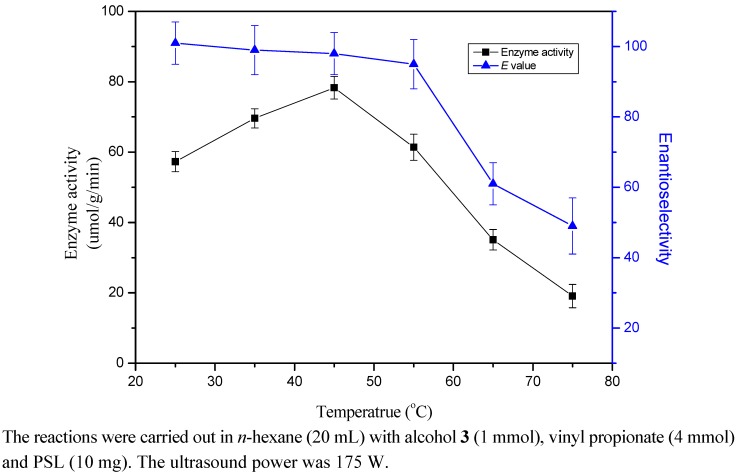
Effect of temperature on the enantioselective transesterification of alcohol **3**.

Regarding the reaction temperature, the optimum temperature for activity and enantioselectivity of PSL for the transesterification of racemic alcohol **3** was screened (25-75 °C) under ultrasound irradiation and the results are shown in Figure 2. The enzyme activity exhibited a bell shaped curve with the changing temperature and the maximal enzyme activity of 78.3 ± 3.2 μmol·g^−1^·min^−1^ was observed at 45 °C. As the reaction temperature is increased, the chance of collisions between enzyme and substrate molecules increases, which might help to form enzyme-substrate complexes and then improve the enzyme activity. The increase of reaction temperature also reduces the viscosity of reaction system and increases the cavitation events under ultrasound irradiation. In enzymatic transesterification, where the enzyme is in a different phase from the substrates, ultrasonic dispersion increases the surface area available to the latter. In essence, cavitation increases the efficiency of the enzyme and optimum activity can be attained. Further temperature increases may destroy the conformation of the enzyme by heat-induced destruction of non-covalent interactions and result in a decrease of enzyme activity. As seen in the results, an increase in temperature (25 °C-55 °C) caused a slight decrease in enantioselectivity, and the *E* values decreased rapidly at higher temperatures (55-75 °C). The phenomenon was in accordance with Sakai who found that lipase exhibited the highest enantioselectivity at low temperatures [30]. It could also be found that the enantioselectivity decreased when the temperature was raised. Indeed, this phenomenon has been reported in a number of cases and a rational understanding of this phenomenon has also been proposed by Phillips [31].

### 2.6. Substrate Ratio

**Figure 3:**
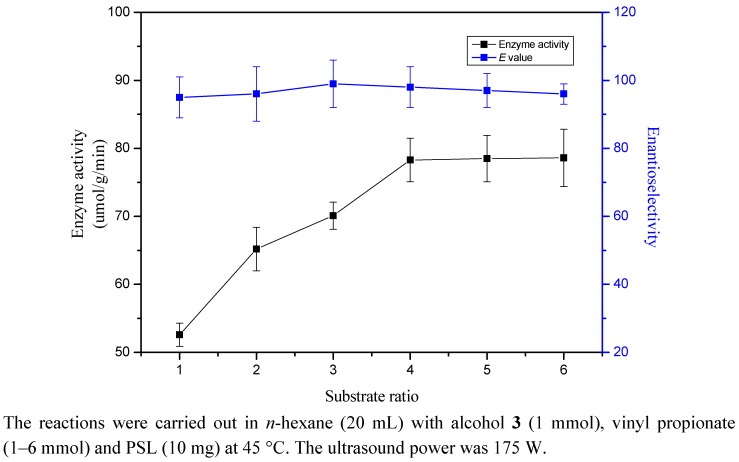
Effect of substrate ratio on the enantioselective transesterification of alcohol **3**.

In this study, the effect of mole ratio of vinyl propionate to racemic alcohol **3** on transesterification when varied from 1:1 to 6:1 was investigated when the amount of the enzyme and alcohol were kept constant. Results are depicted in Figure 3. It was found that the enzyme activity increased with increasing substrate ratio, and the highest activity could be obtained at a 4:1 molar ratio of vinyl propionate to alcohol. Further increasing the substrate ratio did not increase the enzyme activity. The enantioselectivity was not changed by varying the substrate ratios.

## 3. Experimental

### 3.1. Catalysts and Chemicals

*Pseudomonas sp.* lipase (PSL) and *Pseudomonas fluorescens* Lipase (PFL) were purchased from Amano Pharmaceutical Co. Ltd. (Nagoya, Japan). Porcine pancreas lipase (PPL, Lipase type II) was purchased from Sigma (Beijing, China). *Candida antarctica* lipase B (CAL-B) was kindly donated by Novo Nordisk Industries (Beijing, China). *Candida sp. 415* lipase (CSL) was purchased from the Microbial Institute of the Chinese Academy (Beijing, China). Vinyl esters were purchased from Sigma-Aldrich Chemical Co, and were of analytical grade. Other regents were purchased from Shanghai Chemical Reagent Company (Shanghai, China). All the reagents and solvents were used without further purification. (*R*/*S*)-1-chloro-3-(1-naphthyloxy)-2-propanol (**3**) was synthesized in our laboratory according to the reported method [9].

### 3.2. Enantioselective transesterification of (R/S)-1-chloro-3-(1-naphthyloxy)-2-propanol (**3**)

The reaction was performed by using (*R/S*)-1-chloro-3-(1-naphthyloxy)-2-propanol (**3**) (237 mg, 1 mmol), acyl donor (4 mmol), *n*-hexane (20 mL) and lipase (10 mg) with stirring or in an ultrasound bath (175 W) at 45 °C. To determine the conversion of alcohol **3**, the organic samples were withdrawn from the reaction mixture, and analyzed by gas chromatograph (GC). The synthetic activity of enzyme (μmol·g^−1^·min^−1^) was defined as the amount (in micromoles) of alcohol **3**’s ester produced per minute per gram of protein content. The protein content of the used lipases was measured via Lowry method with BSA as a standard for protein concentration [32]. The enantiomeric ratio (*E* value), which dictates the enantioselectivity of the kinetic resolution, was calculated according to Chen *et al.* while the conversion was controlled in the range of the 15~30% [33]:


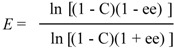


### 3.3. Analytical Methods

The conversion and enantiomeric excess of the remaining substrate was analyzed by gas chromatography (GC) on an Agilent 6890 instrument equipped with a FID detector and an ASTEK ChiraldexTM G-TA (γ-cyclodextrin, trifluoroacetyl) capillary column (30 m × 0.25 mm) with He as the carrier gas (25 mL/min). The temperatures of the injector and detector were 200 and 290 °C, respectively. Temperature programming between 80 and 180 °C with an increment of 10 °C/min was used. Conversions were determined based on the decrease in alcohol, and the ee of the remaining substrate was obtained from the ratio of the peak (*R*)-**3** and (*S*)-**3** area.

## 4. Conclusions

In conclusion, we have reported an effective method for enantioselective tranesterification of (*R*)-1-chloro-3-(1-naphthyloxy)-2-propanol (**3**) catalyzed by lipase under ultrasound irradiation. In this study, the enzyme acitivity and enantioselectivity were increased dramatically under ultrasound irradiation when compared with conventional shaking. The effects of reaction conditions on the synthetic activity of the enzyme as well as enantioselectivity were inverstigated. Under optimum conditions [*n*-hexane (20 mL), alcohol **3** (1 mmol), vinyl propionate (4 mmol), PSL (10 mg), ultrasound power (175 W), temperature (45 °C)], PSL exhibited an excellent catalytic activity and enantioselectivity (enzyme activity: 78.3 ± 3.2 μmol·g^−1^·min^−1^, *E* value: 98 ± 6). Moreover optically pure alcohol (*R*)-3 was obtained (*ee_s_* > 99%) when the conversion reached 52.5%. It is known that immobilization technique may greatly alter the properties of lipases and avoid the enzyme aggregate under ultrasound irradiation [34]. To improve the activity and enantioselectivity of lipase further, a study adopting immobilization techniques is currently in progress and will be reported in due course.

## Notes

*Sample Availability*: Samples of (*R/S*)-1-Chloro-3-(1-naphthyloxy)-2-propanol (**3**) and (*R*)-3 are available from the authors.
